# MAPK Pathways Are Involved in Neuropathic Pain in Rats with Chronic Compression of the Dorsal Root Ganglion

**DOI:** 10.1155/2016/6153215

**Published:** 2016-07-18

**Authors:** Yu-Juan Qu, Lei Jia, Xiao Zhang, Hui Wei, Shou-Wei Yue

**Affiliations:** Department of Physical Medicine & Rehabilitation, Qilu Hospital, Shandong University, Jinan, Shandong 250012, China

## Abstract

The aim of the present study was to investigate whether the MAPK pathways were involved in the mechanism of neuropathic pain in rats with chronic compression of the dorsal root ganglion. We determined the paw withdrawal mechanical threshold (PWMT) of rats before and after CCD surgery and then after p38, JNK, or ERK inhibitors administration. Western blotting, RT-PCR, and immunofluorescence of dorsal root ganglia were performed to investigate the protein and mRNA level of MAPKs and also the alternation in distributions of positive neurons in dorsal root ganglia. Intrathecal administration of MAPKs inhibitors, SB203580 (p38 inhibitor), SP600125 (JNK inhibitor), and U0126 (ERK inhibitor), resulted in a partial reduction in CCD-induced mechanical allodynia. The reduction of allodynia was associated with significant depression in the level of both MAPKs mRNA and protein expression in CCD rats and also associated with the decreased ratios of large size MAPKs positive neurons in dorsal root ganglia. In conclusion, the specific inhibitors of MAPKs contributed to the attenuation of mechanical allodynia in CCD rats and the large size MAPKs positive neurons in dorsal root ganglia were crucial.

## 1. Introduction

Neuropathic pain caused by lesion or inflammation results from the dysfunction and derangement in transmission and signal processing within the nervous system. It is characterized by the symptoms of allodynia, hyperalgesia, and spontaneous pain [1, 2] and it does not depend on the continued presence of tissue-damaging stimuli and is recognized as a disease in itself [3]. Chronic compression of the dorsal root ganglia (CCD) in rats is a typical model of neuropathic pain. During the formation and development of neuropathic pain, inflammation is inevitable. Pain and hyperalgesia that are produced by tissue damage or infection are common features of the inflammatory process [4]. Evidence demonstrates that a substantial proportion of mediators are involved in the symptoms of neuropathic pain, including cytokines, bradykinin, ATP and adenosine, serotonin, eicosanoids, and neurotrophins [1]. Kinds of drugs are used to alleviate neuropathic pain, but they exhibit limited efficacy and undesirable side effects, and neuropathic pain responds poorly to such drug treatments [5].

Mitogen-activated protein kinases (MAPKs), including p38 mitogen-activated protein kinase (p38), c-Jun N-terminal kinase (JNK), and extracellular-regulated kinase (ERK), are a family of serine/threonine protein kinases that transduce extracellular stimuli into intracellular posttranslational and transcriptional responses. A variety of extracellular stimuli activate intracellular MAPKs by phosphorylation, which modulates the intracellular responses that drive different downstream signaling [6]. It is well established that MAPK activation mechanisms are involved in the modulation of nociceptive information and the peripheral and central sensitization produced by intense noxious stimuli [7–12]. Several studies have demonstrated that MAPK pathways play essential roles in inflammation and tissue remodeling [13, 14], and the inhibition of MAPKs produces anti-inflammatory effects in various inflammatory diseases [13]. MAPKs belong to a highly conserved family of serine/threonine protein kinases and are well known to be involved in various aspects of cell signaling and gene expression in the central nervous system (CNS) [15]. MAPKs are thought to be involved in the modulation of inflammation-induced pain hyperalgesia in DRGs and the spinal cord [16]. When the physiopathological mechanisms of inflammatory pain have been studied in patients with amputation neuroma, spinal cord injury, or other causes of neuropathic pain, the mitogen-activated protein kinases (MAPKs) have been found to play a critical role. The phosphorylated forms of these kinases maintain and increase pain signals from the peripheral nociceptors or DRGs by posttranslationally modifying proteins and regulating the transcription of critical genes.

It is demonstrated that specific members of the MAPK family might mediate pain-associated spatial and temporal plasticity in the HF; for example, the local injection of MAPK inhibitors significantly depresses thermal and mechanical hyperalgesia [10, 17–19]. Following peripheral nerve injury, ERK and p38 MAPK are activated and their expression levels are increased in the spinal dorsal horns [7, 10, 20]. There is also evidence supporting that p38 reduces pain by inhibiting p38 phosphorylation via decreasing TNF-*α* [21]. Additionally, JNK signaling plays a crucial role in mediating antinociception and chronic tolerance to the antinociceptive effects of morphine in acute, inflammatory, and neuropathic pain states [22]. The spinal activation mechanisms of MAPK signaling pathways in both neurons and microglia are involved in the antinociceptive effects of pregabalin in a zymosan-induced peripheral inflammatory pain model [23].

Notwithstanding these reports, the underlying role of MAPKs in CCD rats remains unexplored with modern techniques. In the present study, we thus assessed the effects of MAPKs inhibitors in gene and protein expressions and cellular distribution in DRGs and also their effects on allodynia in CCD rats.

## 2. Materials and Methods

### 2.1. Animals and Surgical Procedure

Adult male Wistar rats weighing 180–220 g were provided by the Experimental Animal Center of Shandong University and were housed in a pathogen-free air room at a temperature of 20 ± 2°C at two per cage on a 12 h light/dark cycle with water and food available ad libitum. The animals were allowed 7 days to habituate to the housing prior to manipulation and half an hour to habituate to the experimental environment before every behavioral study was performed. All experimental procedures were approved by the Animal Care and Use Committee of the Shandong University.

Rats were anesthetized by 10% chloral hydrate (300 mg/100 g i.p.), and then two stainless steel rods were implanted unilaterally into the intervertebral foramen at L4 and L5 [24, 25]. Rats in sham-operation group underwent the same operation but with no steel bar insertion. The rats with autophagy phenomenon, feeling deficiency, and disability were eliminated.

### 2.2. Behavioral Testing

Behavioral testing was performed using the ipsilateral hind paw of the animals prior to the operation, on postoperative day 4, and 2 hours after the injection of inhibitors. The paw withdrawal mechanical thresholds (PWMTs) were evaluated with a BME-404 Mechanical Analgesia Tester (CAMS-Chinese Academy of Medical Sciences, Beijing, China) [25]. The probe was pressed against the lateral plantar surface of the hind paw with sufficient force. A positive response was noted when the paw was immediately withdrawn. The rats were tested again at least five minutes later, the tests were repeated five times, and the average was calculated and used in the statistical analyses.

### 2.3. Western Blot Analysis

Four days after surgery, CCD rats were intrathecally injected with MAPKs inhibitors for 2 h. The L4 and L5 ganglia from the operated side were quickly and carefully harvested. The samples of total protein were separated by sequential 5% and 10% SDS-PAGE and then transferred to polyvinylidene fluoride membranes. The membranes were incubated in 5% milk for 2 h at room temperature. Next, the membranes were incubated with primary antibody at 4°C overnight and subsequently with horseradish peroxides- (HRP-) conjugated secondary antibodies for 1 h. The signals were detected with Immobilon*™* Western Chemiluminescent HRP Substrate. The primary antibodies were rabbit anti-ERK polyclonal antibody (1 : 1,000, CST, USA), rabbit anti-p-ERK polyclonal antibody (1 : 2,000, CST, USA), rabbit anti-JNK polyclonal antibody (1 : 1,000, CST, USA), rabbit anti-p-JNK polyclonal antibody (1 : 1,000, CST, USA), rabbit anti-p38 polyclonal antibody (1 : 200, CST, USA), and rabbit anti-P-p38 polyclonal antibody (1 : 1,000, CST, USA). The second antibody was goat-anti-rabbit antibody (1 : 8,000, Zhongshan Golden Bridge, Beijing, China). The protein bands were developed with a FluoroChem 9900 imaging system (USA), and the quantifications of the intensities of the bands were performed with the Quantity One software and normalized to *β*-tubulin (1 : 1,000, CST, USA).

### 2.4. Immunolocalization of p38, ERK, and JNK in Dorsal Root Ganglia

Rats were deeply anesthetized with 5% isoflurane and perfused transcardially with cold normal saline followed by fixative containing 4% paraformaldehyde in 0.1 M phosphate-buffered saline (PBS, pH 6.9). The ipsilateral lumbar L4-L5 DRGs were removed rapidly after perfusion, postfixed in the same fixative overnight at 4°C, and then dehydrated and paraffin-infused. A series of paraffin sections (4 *μ*m) were cut using a rotary microtome. The sections were incubated separately in mixtures of the following primary antibodies at 4°C overnight: rabbit anti-ERK polyclonal antibody (1 : 200, CST, USA), rabbit anti-JNK polyclonal antibody (1 : 200, CST, USA), and rabbit anti-p38 polyclonal antibody (1 : 50, CST, USA). The primary antibodies were combined with mouse-anti-NF200 polyclonal antibody (1 : 1,000, Abcam, Cambridge, UK). Then the sections were incubated in Alexa Fluor 488-conjugated Affinipure Donkey Anti-Rabbit IgG (H+L) and Alexa Fluor 594-conjugated Donkey Anti-Mouse IgG (H+L) for 2 h at room temperature. DAPI was used to stain the cell nuclei.

Labeled sections were examined under an Olympus-u-rfl-t/dp 72 automatic fluorescence microscope using the image analysis system of the microscope (JA) and analyzed using the IPP.6 software. For the quantitative analyses of the numbers of positive neurons, three immunofluorescent stained nonconsecutive sections were imaged per ganglion. The data were collected from three animals for each inhibitor (SB203580, U0126, and SP600125).

### 2.5. Real-Time Quantitative RT-PCR

L4 and L5 ganglions were harvested in the same manner as described above. Fragments of p38, JNK, ERK, or *β*-actin were amplified with the following primers: p38 (forward, 5′-CCTGCGAGGGCTGAAGTA-3′; reverse, 5′-ACGGACCAAATATCCACTGTCT-3′), JNK (forward, 5′-AGCCTTGTCCTTCGTGTC-3′; reverse, 5′-AAAGTGGTCAACAGAGCC-3′), ERK1 (forward, 5′-CCAGAGTGGCTATCAAGAAG-3′; reverse, 5′-TCCATGAGGTCCTGAACAA-3′), ERK2 (forward, 5′-TGCCGTGGAACAGGTTGT-3′; reverse, 5′-TGGGCTCATCACTTGGGT-3′), and *β*-actin (forward, 5′-AGACCTTCAACACCCCAG-3′; reverse, 5′-CACGATTTCCCTCTCAGC-3′). Instrument control, automated data collection, and data analysis were all performed using the Light Cycler software program, version 4.0. The 2^−ΔΔCT^ method was used to analyze the data.

### 2.6. Chemicals and Reagents

The following chemicals were used in this study: SB203580 (p38 inhibitor, CST, USA, recommended concentration = 40 *μ*mol/L), SP600125 (JNK inhibitor, CST, USA, recommended concentration = 50 *μ*mol/L), and U0126 (ERK inhibitor, CST, USA, recommended concentration = 40 *μ*mol/L). All of the chemicals were dissolved in DMSO, and the final experimental dilutions were made in normal saline on the day of the experiment.

### 2.7. Data Analysis and Statistics

All calculations and statistical analyses were performed using Prism 5.0 (Graph Pad Software, San Diego, CA, USA). A two-way repeated measures ANOVA was used to analyze the differences in the PWLs, the level of protein and gene expression, and the neurons distribution. Values in the test were expressed as means ± SDs. *P* values < 0.05 were considered significant.

## 3. Results

### 3.1. PWMT Changes after the CCD Operation

**Figure 1:**
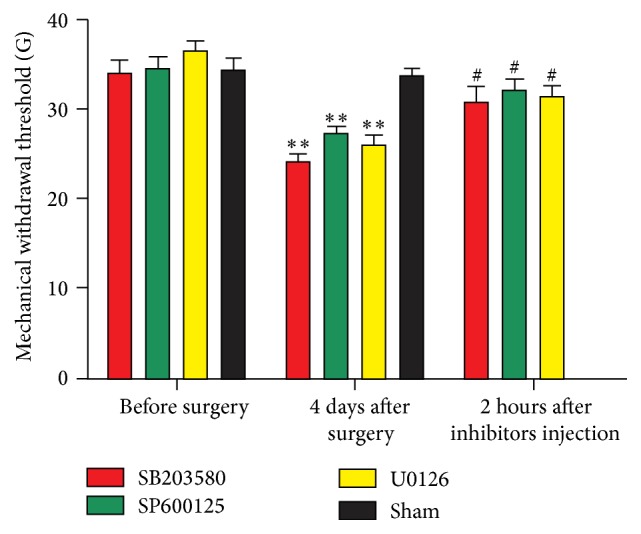
Alternations in PWMTs. ^*∗∗*^ *P* < 0.01 compared with control group; *n* = 8 in each group; ^#^ *P* < 0.05 compared with the CCD groups; *n* = 8 in each group.

To detect whether the inhibitors of MAPKs attenuated CCD-induced neuropathic pain, PWMTs were examined before surgery, 4 days after surgery, and 2 h after inhibitors administration. As shown in Figure 1, the CCD group developed evident mechanical allodynia hyperalgesia in the ipsilateral hind paw compared with the control group. The PWMT significantly decreased at 4 days after the CCD operation (*n* = 8 in each group; ^*∗∗*^ *P* < 0.01). Furthermore, CCD-induced allodynia was attenuated by SB203580, SP00125, and U0126 (*n* = 8 in each group; ^#^ *P* < 0.05), while there was no significant difference between sham group and control group (*n* = 8 in each group).

### 3.2. Changes in Protein Expressions of p38, ERK, and JNK in the DRGs

**Figure 2:**
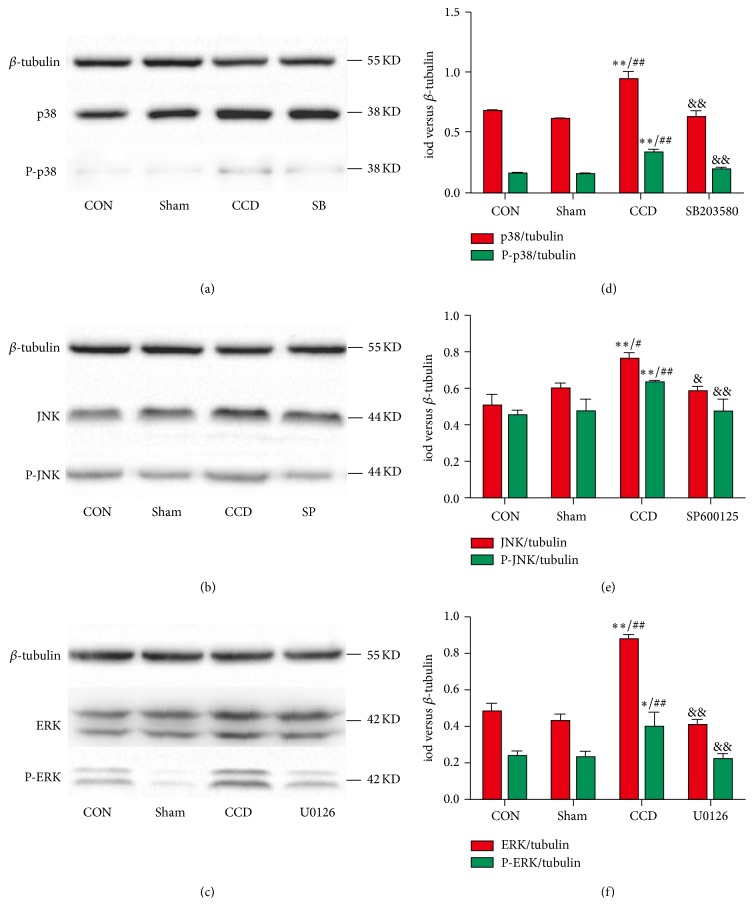
Effects of the inhibitors on protein expressions of p38, JNK, and ERK and their phosphorylation. (a)–(c) show the expressions of p38, P-p38, JNK, P-JNK, ERK, and P-ERK after SB203580, U0126, and SP600125 administration, and (d)–(f) show the iods compared with *β*-tubulin. ^*∗*^ *P* < 0.05, ^*∗∗*^ *P* < 0.01, and *n* = 5 for each group compared with the control group; ^#^ *P* < 0.05 and ^##^ *P* < 0.01 compared with the sham group; *n* = 5 in each group; ^&^ *P* < 0.05 and ^&&^ *P* < 0.01 compared with the CCD group; *n* = 5 in each group.

To investigate whether p38, ERK, and JNK expression and phosphorylation were altered, pharmacological inhibitors were administered to the CCD rats. As demonstrated in Figure 2, the levels of p38, JNK, ERK, P-p38, P-JNK, and P-ERK protein expression in the CCD rats significantly increased (*n* = 5, ^*∗*^ *P* < 0.05, and ^*∗∗*^ *P* < 0.01 compared with control group; ^#^ *P* < 0.05 and ^##^ *P* < 0.01 compared with sham group). These CCD-induced increases in protein expression level were diminished significantly by inhibitors (SB203580, SP600125, and U0126) administration (^&^ *P* < 0.05 and ^&&^ *P* < 0.01 compared with CCD groups).

### 3.3. Changes in Gene Expressions of p38, ERK, and JNK in the DRGs

**Figure 3:**
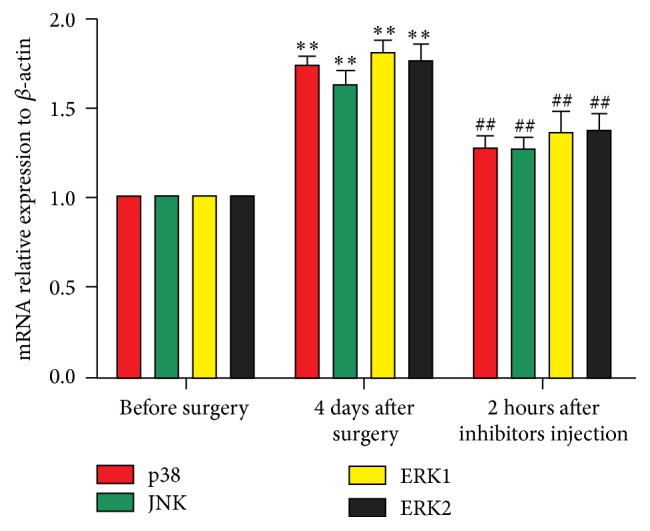
Relative expression levels of p38, JNK, and ERK mRNA in DRGs. *n* = 6 in each group. ^*∗*^ *P* < 0.05 and ^*∗∗*^ *P* < 0.01 compared with the normal rats. ^#^ *P* < 0.05 and ^##^ *P* < 0.01 compared with CCD rats.

Pharmacological inhibitors of MAPKs not only diminished the protein expression of p38, ERK, and JNK in DRGs of CCD rats but also affected the level of gene expressions. As demonstrated in Figure 3, the levels of p38, JNK, and ERK gene expression in the CCD rats significantly increased (*n* = 6 and ^*∗*^ *P* < 0.05 and ^*∗∗*^ *P* < 0.01 compared with control group). These CCD-induced increases in gene expression level were diminished significantly by inhibitors (SB203580, SP600125, and U0126) administration (*n* = 6 and ^##^ *P* < 0.01 compared with CCD groups).

### 3.4. Changes in p38, ERK, and JNK Distributions in DRG Neurons

To quantify the proportions of positive cells within defined subsets of sensory neurons, we counted the numbers of positive neurons detected in the NF200-immunoreactive neuronal profiles.

**Figure 4:**
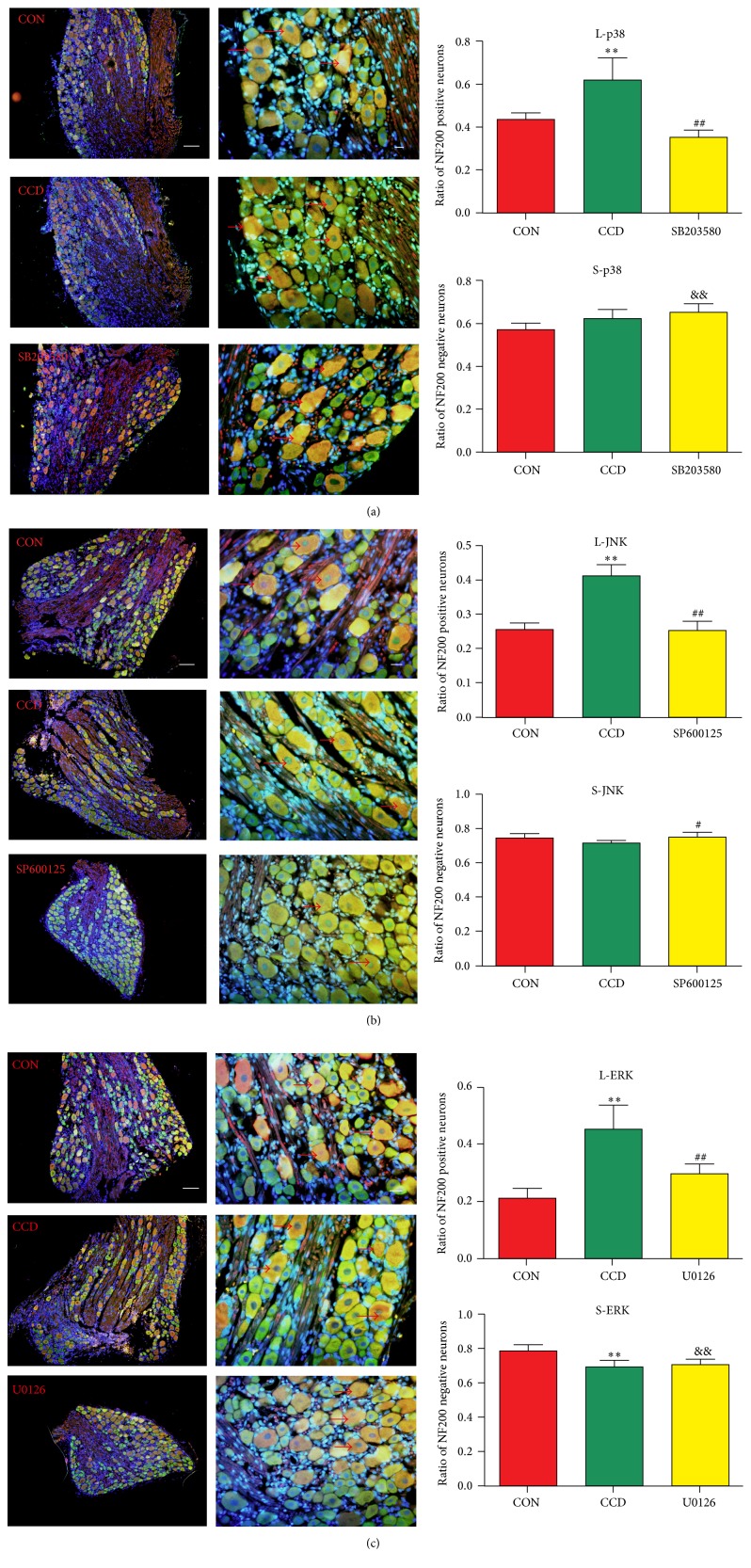
Changes in the distributions of p38, JNK, or ERK/NF200 positive or negative neurons in the DRG tissue. (a)–(c) illustrate the neuronal distributions and ratios of the NF200 positive or negative neurons. *N* = 6; ^*∗*^ *P* < 0.05 and ^*∗∗*^ *P* < 0.01 compared with the control group; ^#^ *P* < 0.05 and ^##^ *P* < 0.01 compared with the CCD group and ^&&^ *P* < 0.01 compared with the control group. Scale bars: 100 *μ*m for the low-power field and 20 *μ*m for high-power field (→: NF200 positive neurons).

As demonstrated in Figures 4(a)–4(c), p38, JNK, and ERK were expressed in both the nuclei and cytoplasm; the proportions of NF200 positive large size neurons among all of the neurons in the DRG tissues increased significantly (*n* = 6 and ^*∗*^ *P* < 0.05 and ^*∗∗*^ *P* < 0.01) after CCD surgery compared with control groups. After SB203580, SP600125, or U0126 administration, the proportions of NF200 positive neurons significantly decreased (*n* = 6 and ^#^ *P* < 0.05 and ^##^ *P* < 0.01 compared with CCD group). As to NF200 negative small size neurons, though there were some changes in proportion, we could not find any obvious regulation of these changes.

## 4. Discussion

This is the first study showing the role of MAPK pathways in neuropathic pain in DRGs of CCD rats. Intrathecal administration of the MAPKs inhibitors, SB203580, SP600125, and U0126, resulted in a partial reduction in CCD-induced mechanical allodynia. The reduction of allodynia was associated with significant depression in the level of both MAPKs gene and protein expression in CCD rats, and the large size MAPKs positive neurons in dorsal root ganglia were crucial in maintaining the neuropathic pain.

The CCD model has been proven to be a typical model of neuropathic pain. In CCD rats, the direct mechanical compression of the DRG [26] and secondary inflammatory processes [27] induce hyperexcitability of the DRG neurons, and this hyperexcitability is associated with allodynia and changes in the lower paw withdrawal latency after CCD surgery. Using specific inhibitors of MAPKs contributed to the attenuation of mechanical allodynia in CCD rats. MAPK pathways may be new targets of neuropathic pain treatment.

In the CNS, the activation of the p38 MAPK pathway constitutes a key step in the development of neuroinflammation. Inflammatory stimuli bind to receptors on the cell surface to trigger intracellular signal transduction pathways, such as the p38 MAPK pathways [28, 29]. Subsequently, intracellular p38 MAPK is activated and profoundly modulates somatic inflammatory responses. The expression of ERK in the DRGs has been implicated in the induction of neuropathic pain behaviors in rat models of chronic constriction injury (CCI) and the normalization of those behaviors after decompression of the CCI reflects the reversal of the pain behaviors [30]. The expression of JNK is also activated in the spinal DRG after nerve injury, and this expression of p-JNK can maintain mechanical allodynia [31]. Inhibitors of MAPKs administration in recommendatory dose and time specifically decreased the upregulated protein expression of MAPKs (p38 and P-p38 by SB203580, JNK and P-JNK by SP600125, and ERK and P-ERK by U0126) in CCD rats. Similar changes were found in the gene expression of p38, JNK, and ERK. However, the dose/time dependence of these inhibitors may require further analysis.

Neurons in DRGs are divided into three types (large: >35 *μ*m with A*β* fiber; middle: 20–35 *μ*m with A*δ* fiber; small: <20 *μ*m with C fiber), mainly depending on their size, electrophysiological property, and neuronal processes [32]; generally, A*β* fiber conducts proprioception and tactile sense, C fiber conducts nociception signal, and A*δ* fiber conducts both. The proportions of NF200 positive large size neurons among the p38, JNK, or ERK positive neurons in the DRG tissues increased significantly after CCD surgery; then the proportions were decreased by SB203580, SP600125, or U0126 administration, while the proportions of NF200 negative small size neurons change without explicable regulation. Therefore, the large size neurons with A*β* fiber contributed mainly to the MAPKs mediated neuropathic pain in CCD rats.

## 5. Conclusions

In conclusion, the present study demonstrated that specific inhibitors of MAPKs contributed to the attenuation of mechanical allodynia in CCD rats and the large size MAPKs positive neurons in dorsal root ganglia were crucial. Therefore, MAPK pathways are involved in the mechanism of neuropathic pain in CCD rats.
